# Facile Hydrothermal Assisted Basic Catalyzed Sol Gel Synthesis for Mesoporous Silica Nanoparticle from Alkali Silicate Solutions Using Dual Structural Templates

**DOI:** 10.3390/gels10120839

**Published:** 2024-12-19

**Authors:** Khaled M. AlMohaimadi, Hassan M. Albishri, Khaled A. Thumayri, Awadh O. AlSuhaimi, Yassin T. H. Mehdar, Belal H. M. Hussein

**Affiliations:** 1 Chemistry Department, Faculty of Science, King Abdulaziz University, P.O. Box 80203, Jeddah 21589, Saudi Arabia; khaled-mohaimadi@hotmail.com; 2 Chemistry Department, Faculty of Science, Taibah University, Medina Munwarah 42353, Saudi Arabia; kthumairi@taibahu.edu.sa (K.A.T.); asuhaimi@taibahu.edu.sa (A.O.A.); ymehdar@taibahu.edu.sa (Y.T.H.M.); belalhussein102@yahoo.com (B.H.M.H.); 3 Department of Chemistry, Faculty of Science, Suez Canal University, Ismailia 41522, Egypt

**Keywords:** mesoporous silica particles, alkali silicate solution, PEG/CTAC dual templates, adsorption, cationic dyes

## Abstract

This work presents a novel hydrothermally aided sol-gel method for preparation of mesoporous silica nanoparticles (MSNs) with a narrow particle size distribution and varied pore sizes. The method was carried out in alkaline media in presence of polyethylene glycol (PEG) and cetyltrimethylammonium chloride (CTAC) as dual templates and permitted the synthesis of spherical mesoporous silica with a high surface area (1011.42 m^2^/g). The MSN materials were characterized by FTIR, Thermogravimetric (TG), Nitrogen adsorption and desorption and Field emission scanning electron microscopic analysis (FESEM). The materials feasibility as solid phase adsorbent has been demonstrated using cationic dyes; Rhodamine B (RB) and methylene blue (MB) as models. Due to the large surface area and variable pore width, the adsorption behaviors toward cationic dyes showed outstanding removal efficiency and a rapid sorption rate. The adsorption isotherms of RB and MB were well-fitted to the Langmuir and Freundlich models, while the kinetic behaviours adhered closely to the pseudo-second-order pattern. The maximum adsorption capacities were determined to be 256 mg/g for MB and 110.3 mg/g for RB. The findings suggest that MSNs hold significant potential as solid-phase nanosorbents for the extraction and purification of dye pollutants, particularly in the analysis and treatment of effluents containing cationic dyes.

## 1. Introduction

Mesoporous silica nanoparticles (MSNs) have experienced considerable development from their early beginning, which traces back to the discovery of colloidal silica particles suspended in liquid media. Colloidal silica was first identified in the early 20th century [1,2] and was investigated for its stability and capacity to form sols [3,4]. In 1968, Werner Stöber and his coworkers accomplished an important breakthrough by devising a simple hydrolysis condensation technique that enabled the controlled synthesis of monodisperse spherical silica particles from tetraethyl orthosilicate (TEOS) in an aqueous solution containing ammonia and alcohol [5]. In the early 1990s, researchers synthesised the first mesoporous materials, including Mobil Composition of Matter No. 41 (MCM-41) and Santa Barbara Amorphous-15 (SBA-15) and others, which exhibited hexagonal arrangements of mesopores with uniform pore diameters, utilising sol-gel chemistry in conjunction with architectural templates such as surfactants [6,7,8,9,10]. The initial development of MSNs was driven by applications requiring large surface area as well as controlled nanoscale dimensions. The synthesis of MSNs is achieved by merging templated techniques used in the fabrication of mesoporous materials with methods employed in the preparation of colloidal silica particles [11,12,13,14]. Initially, the development of MSNs attracted significant interest for biological applications such as drug delivery, imaging, and diagnostics, owing to their large surface area, tuneable pore size, and ability to carry a wide variety of therapeutic agents [15,16,17,18]. Beyond biological applications, MSNs have also been employed in environmental remediation [19,20], catalysis [21], and energy storage [22], leveraging their unique and versatile properties.

MSNs are commonly synthesised from alkoxysilane precursors, with tetraethyl orthosilicate (TEOS) being a popular choice for its capacity to generate consistent, pure, and meticulously regulated nanoparticles. The synthetic process entails acid- or base-catalysed hydrolysis and condensation of alkoxysilane precursors in aqueous or alcoholic mediums [6,7,8,23]. Structural directing agents (SDAs) like cetyltrimethylammonium bromide (CTAB) and macromolecules such as polyethylene glycol (PEG) are added to the mixture to create mesopores during the sol-gel process. After synthesis, the SDAs are typically eliminated through either solvent extraction or calcination to maintain the mesoporous configuration [23,24,25].

The high costs and environmental concerns associated with alkoxysilane-based precursors have driven the search for more sustainable alternatives. Among these, sodium and potassium silicates—readily available alkali silicate solutions—have emerged as attractive options due to their affordability and scalability [26,27]. Many innovative methods have been developed to synthesize MSNs using these eco-friendly silica sources. Notable examples include the utilization of silica-rich agricultural byproducts, such as rice husk ash and bagasse, as well as various industrial wastes [28,29,30]. By employing alkali silicate precursors, MSN synthesis not only reduces production costs but also promotes environmentally responsible practices in nanomaterial fabrication.

The synthesis typically entails mixing diluted alkali silicate solutions with SDA which function as templates to facilitate the development of homogeneous mesopores, followed by controlled hydrolysis and condensation processes under mild conditions [27,28,29,30,31]. One of the earliest attempts to fabricate mesoporous silica particles was reported by Sierra et al. Those authors demonstrated a simple procedure for the synthesis of mesoporous particles from reaction mixtures containing sodium silicates (as a source of silicic acids), sodium chloride, and a non-ionic surfactant (Triton X100) at temperatures ranging from 25 °C to 45 °C. It has been observed that particle size reduced from several tens of micrometres to below 1 µm (~500 nm) as the pH increased from 1.85 to 6 [30]. Lin et al. pioneered a kinetics-based approach for synthesising nanoscale mesoporous silica from diluted surfactant-sodium silicate solution with reduced nanoparticle aggregation [26]. Sodium silicate and alkyltrimethylammonium halide surfactant were dissolved in water to make a transparent solution at pH 11–12 and 40 °C. A sulphate or acetate acid aqueous solution is added to lower the pH to 5.5–9.0. The obtained MSNs had particle sizes 30–100 nm, pores 2.08–2.71 nm, and surface areas 760–1122 m^2^/g. In another sol-gel method, polyethylene glycol (PEG) was utilized as a templating agent instead of traditional surfactants. This approach allowed the fabrication of MSNs with pores ranging from 3.3 to 3.9 nm. Following the template removal process via calcination and solvothermal extraction, MSNs with a remarkable surface area of 634 m^2^/g were successfully generated. The synthesis process involved the addition of a diluted sodium silicate solution (8.0%) into an acidic medium (1 M HCl) containing PEG under intense stirring. The pH level was carefully maintained at 4 using NaOH. A similar method was employed to create MSNs from a sodium silicate solution with PEG as a template [32]. Initially, the sodium silicate solution was derived from bagasse ash by its dissolution in NaOH. The resulting material exhibited a surface area and pore diameter of approximately 525–656 m^2^/g and 18 nm, respectively. The authors employed three techniques to induce gel formation: the addition of HCl solution, mixing with acidic ion exchange resin, and incorporating an acidic PEG solution. PEG was removed to produce porous silica through calcination and/or solvothermal extraction [33]. Hwang et al. developed spherical MSNs from a sodium silicate solution using a sol-gel method, employing PEG as a SDA and acetic acid as a pH modifier. The resulting materials displayed surface areas ranging from 109 to 685 m^2^/g, with pore sizes varying from 3 to 40 nm [27].

Sol-gel procedures can be carried out at ambient or moderate temperatures (e.g., 50–80 °C); however, in some applications, hydrothermal-assisted sol-gel synthesis is preferred because it provides better control over MSN size, shape, and pore structure due to regulated temperature and pressure. Hydrothermal treatment enhances nanoparticle crystallinity and stability while improving surface area and pore distribution. This approach also enhances MSN’s mechanical strength and thermal stability [34,35,36,37].

A recent work by Tella et al. [38] investigated the textural features of amino-functionalized MSNs synthesised from TEOS and sodium silicate solution precursors by hydrothermal-assisted sol-gel methods, utilising CTAB as an SDA template. The results showed that the MSNs derived from sodium silicate solution exhibited a sheet-like morphology, which decomposed after calcination, leading to the formation of an agglomeration of small spherical particles. Furthermore, the mesoporosity of the particles exhibited a lower degree of organisation in comparison to the TEOS-derived materials. The application of PEG as a template during MSN synthesis was shown to have a significant effect on particle size. When used in a ratio of 2:1 compared to sodium silicate solution, the particle size was dramatically reduced from 250 to 60 nm, and the size distribution of nanoparticles improved [39].

The synthesis of MSNs from alkali silicate through the sol-gel process is predominantly an acid-catalysed method, in which acid is employed to modulate the pH to an acidic or neutral range [35,36,37,38,39]. Interestingly, Zulfiqar et al. have synthesised non-agglomerated silica nanoparticles from alkali silicate solution precursors in an alkaline medium counting NH_4_OH and ethanol [40]. Nevertheless, the synthesis of colloidal silica has been reported via the method of passing a diluted alkali silicate solution through an ion-exchange resin, subsequently titrated with potassium hydroxide [41]; however, the particle size distribution remained poorly defined [42]. It is believed that at this pH, silicate anions are effectively solubilised, facilitating the condensation of silica into a mesoporous structure in the presence of templating agents such as surfactants. A relatively basic environment facilitates the equilibrium of nucleation and growth rates of silica framework, resulting in well-defined mesoporous structures. Given that sodium silicate solution is highly basic (pH > 12), the pH must be lowered with acid prior to conducting condensation polymerisation in a basic medium. This may cause uncontrollable gelation and/or introduce impurities from acid and basic solutions.

In this study, a cation exchanger was utilized to remove sodium ions and convert the silicate solution into active silicic acid (Si(OH)_4_) at a pH of 4–6. This activated solution was subsequently introduced into a mixture containing CTAC and PEG as dual SDAs, along with NH_4_OH as a catalyst to facilitate the sol-gel process at a pH of 10.5. As a proof of concept, the synthesized MSNs were applied as nano-adsorbents for the extraction of dyes from synthetic wastewater samples. Notably, this is the first study to utilize hydrothermal assistance combined with dual SDAs (PEG and CTAC) to synthesize MSNs with a large surface area and narrow pore size distribution. These properties make the resulting MSNs an ideal sorbent, demonstrating high efficiency in dye extraction and removal from water bodies.

## 2. Results and Discussion

### 2.1. Chemistry and Characterization of MSNs

**Figure 1:**
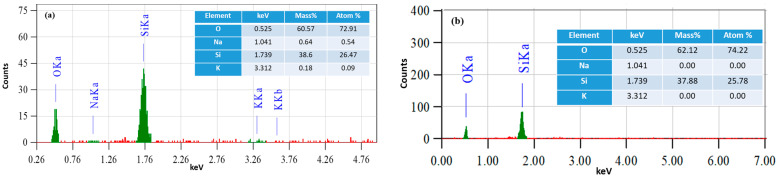
A comparison of EDS data (**a**) before and (**b**) after ion exchange.

MSNs with varied sizes and surface areas were synthesized from sodium silicate solution using the proposed sol-gel process catalyzed by ammonia, utilizing CTAC and PEG as dual templates at different molar ratios. MSNs having a large surface area and smaller average diameter were synthesized utilizing different PEG/CTAC molar ratios (0.5, 1, 1.25, 1.5, and 2) and constant concentrations of sodium silicate, NH_4_OH, and water. The hydrothermal condition at 120 °C is essential, while mesoporous channels become more distinct at elevated temperatures, signifying enhanced pore structure ordering. As the temperature increased, the dimensions of the nanospheres enlarged and achieved greater uniformity. Heating at higher temperatures promoted the hydrolysis and polymerization rates of sodium silicate solution, allowing the synthesis of monodispersed MSN. Cation exchange is an excellent method for producing active silicic acid, as the gelation process accelerates with a lower pH [43]. It also reduces the concentrations of Na^+^ and K^+^ ions in the produced MSNs, as these ions have been replaced with H^+^ ions. The energy dispersive X-ray spectroscopy (EDS) analysis of MSNs synthesized from sodium silicate solutions, both prior to and after cation exchange, demonstrated that the sodium and potassium ions were removed throughout the ion exchange process (Figure 1).

The formation of the MSNs is proposed to involve several steps: Initially, PEG is incorporated into CTAC micelles to create a self-assembled spherical template structure at crucial micelle concentrations. It has been indicated that as the mole ratio of PEG to CTAC increased, so did the pore size (Table 1). Ammonia-catalyzed sodium silicate hydrolysis generates negatively charged oligomeric silicate species that interact electrostatically with the PEG/CTAC micelle surface to form a spherical CTAC-PEG silicate complex. CTAC and PEG concentrations have a significant influence on average particle size and agglomeration, while PEG molecules’ steric repulsion restricts nanoparticle formation. MSNs are thought to originate by a crystal growth mechanism that includes self-assembly and the layered production of spherical or pseudospherical micelles of varying sizes. Sierra and Rodenas [44] confirmed that poly (propylene glycol) interacts with CTAB micelles, resulting in CTAB/PPG mixed micelles. Incorporating polymers into CTAB micelles increases micellar ionization while decreasing surfactant aggregation and critical micelle concentration (cmc) values. These effects are consistent with medium-chain alcohols. When oppositely charged polymers and surfactants are mixed, they can generate a concentrated phase rich in micelles linked together by polymer chains, yielding nanoparticles of aggregated micelles joined by polyion chains. It has been shown that the variations of polymer chain length had no influence on morphology but reduced the diameter of nanoparticles [45]. The interactions between cationic surfactant head groups and PEG produce spherical micelles, which are subsequently partially hydrolyzed and condensed to create a thin silica shell. The interaction of surfactant micelles with silica species greatly influences the structure of mesoporous silica. The geometry of surfactant micelles is known to have a substantial influence on the morphology of mesoporous materials.

**Figure 2:**
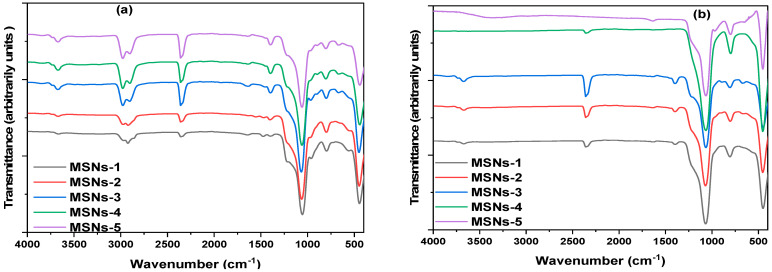
The infrared spectrum of mesoporous silica nanoparticles with CTAC/PEG composites (**a**) and after removal of CTAC and PEG molecules (**b**).

**Figure 3:**
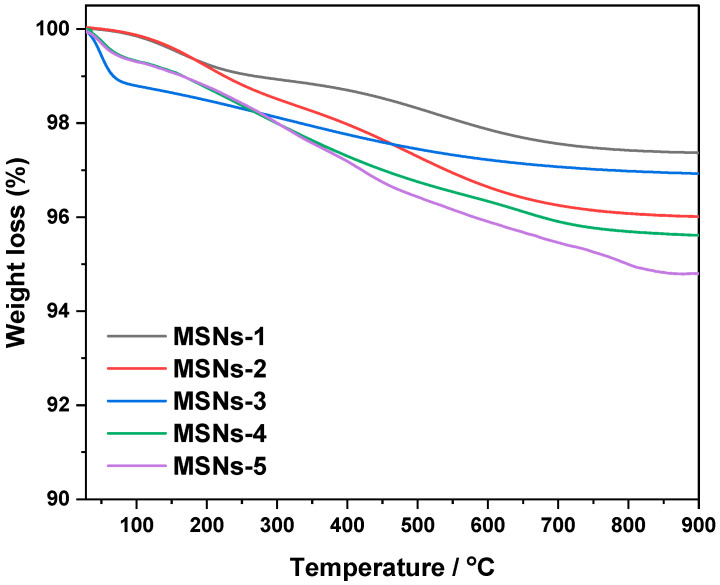
Thermal analysis profiles of mesoporous silica nanoparticles after removal CTAC and PEG molecules.

Since the removal of CTAC surfactant and PEG is crucial before MSN characterization and applications, the materials were calcinated at 550 °C. The effectiveness of calcination was monitored using FTIR spectra (Figure 2) and thermogravimetric (TG, Figure 3) measurements of the MSNs to insure that the CTAC and PEG were removed satisfactorily. Figure 2 shows the typical FTIR spectra of MSNs produced with CTAC/PEG templating and CTAC/PEG-free silica after CTAC/PEG removal. The FTIR spectra of silica-CTAC/PEG revealed two silica bands at 1070 and 800 cm^−1^, corresponding to Si-O-Si bonds. The 960–952 cm^−1^ band is formed by the stretching of non-bridging oxygen atoms, such as Si-O- [46]. Figure 2a shows that the bands at 2977 and 2892 cm^−1^ correspond to the alkyl group (-CH_2_-) of CTAC and PEG molecules [47,48]. The signals indicate the conversion of CTAC and PEG into silica particles. The vibration symmetry band of N-CH_3_ feature shows a slight shoulder at 2970 cm^−1^ and a very weak band at 2890 cm^−1^. The detection of (-N-CH_3_) and (C-N) bands of CTAC at 1396 ± 50 and 912 ± 25 cm^−1^ is consistent with previous literature findings. It is crucial to note that the bands between 1400 and 900 cm^−1^, attributed to methyl rocking and C-N stretching modes of -N^+^(CH_3_)_3_ groups, are difficult to distinguish due to overlap with MSNs bands in the same area [49,50,51]. The lack of CTAC and PEG peaks (Figure 2b) such as C-H (2977–2890 cm^−1^) proved that the surfactant of the produced mesoporous silica had been removed. The bands at 2350 ± 30 correspond to locations with different hydrogen bonding strengths with nonbridging oxygens. Scholze assumed that the hydroxyl groups alone are responsible for the absorption in the 2300–3700 cm^−1^ range [52,53]. Notably, the bands between 2000 and 2500 cm^−1^ are caused by carbon dioxide adsorbed/entrapped within mesopores from the environment or adsorbed during calcination.

TG curves for calcined silica materials revealed weight loss below 180 °C, indicating the removal of physically adsorbed water (Figure 3). Weight loss maxima were seen between 400 and 600 °C, which might be attributed to the dehydration of silanol groups on the MSN surface. Thus, it was demonstrated that PEG and CTAC were effectively removed from MSNs.

**Figure 4:**
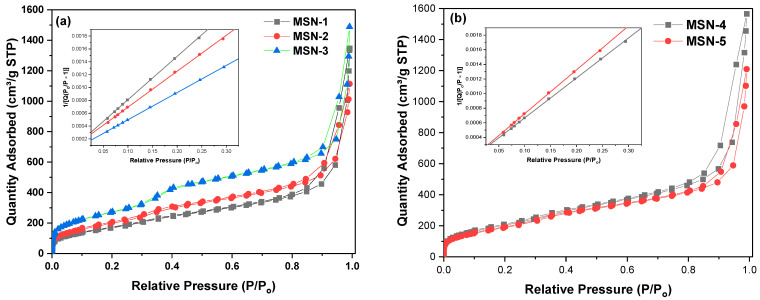
N_2_ adsorption-desorption isotherm curves of MSNs. Inset show the linear ship of relative pressure. (**a**) MSN-1 (black), MSN-2 (red), MSN-3 (blue); (**b**) MSN-4 (black), MSN-5 (red).

**Figure 5:**
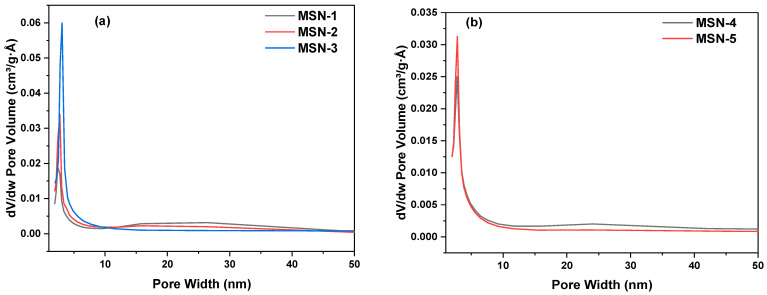
BJH pore size distribution plots of MSNs. (**a**) MSN-1 (black), MSN-2 (red), MSN-3 (blue); (**b**) MSN-4 (black), MSN-5 (red).

The concentration of CTAC and PEG has a substantial influence on surface area, average particle size, and agglomeration, however the steric repulsion generated by PEG molecules restricts nanoparticle growth. More importantly, MSN formation is prolonged, which significantly improves both sphericity and monodispersity. It is critical to note that the time intervals between adding sodium silicate solution must be sufficient to consume the majority of residual silicate species; otherwise, the local over-concentration of silicate species would result in significant accumulation and agglomeration between the silica surfactant micelles, and eventually between MSNs [54]. N_2_ adsorption and desorption isotherms (BET) may be utilized to calculate MSN surface area. After several trials, the following criteria were established: CTAC and PEG removal must be finished, MSNs thoroughly cleaned with water and ethanol, and the final ethanol solution evacuated at reduced pressure. N_2_ adsorption/desorption tests on MSNs yielded type IV isotherms with a prominent H1 hysteresis loop at high relative pressure, which is typical of mesoporous materials as seen in Figure 4 [55,56]. The PEG/CTAC as dual template significantly increased the surface area of MSNs, reaching roughly 1011 m^2^g^−1^ after removal the organic template. The surface area increased with the PEG/CTAC molar ratio, peaked, and subsequently (Table 1) as the PEG concentration increased. MSNs at high PEG concentration a densification of pore structure resulting a smaller surface area pore diameter and pore volume. BJH adsorption cumulative volume pore values recorded for MSNs were varied from 1.73 cm^3^g^−1^ to 2.37 cm^3^g^−1^. Figure 5 shows the pore size distribution of porous silica prepared by calcination at different PEG concentrations. The distribution is narrow at high and low PEG concentration, indicating the pore size of silica is not significantly influenced by PEG concentrations under experimental conditions in the presence CTAC as co surfactant. MSN present a very narrow pore size distribution centered at 3.1 nm at 0.54 mM PEG concentration (Table 1). PEG interacts significantly with silanols, influencing the polycondensation of the silicic acid species. At low PEG concentrations, a synergetic interaction between PEG and silica occurs in which negatively charged silica drives PEG aggregation, resulting in a gel network that directs the formation of silica particles. Thus, PEG was trapped in silica networks and used as a template, resulting in pores following extraction. At high PEG concentrations, however, silica addition produced a PEG-silica precipitate, which was followed by the formation of gel embedded in the composite precipitate. As a consequence, increasing the PEG content had no significant effect on the surface area or pore volume.

**Figure 6:**
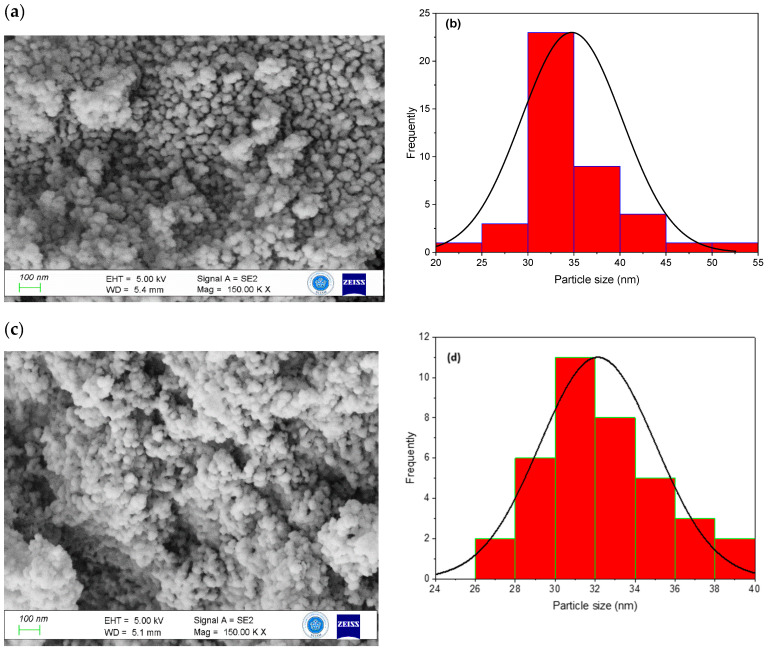

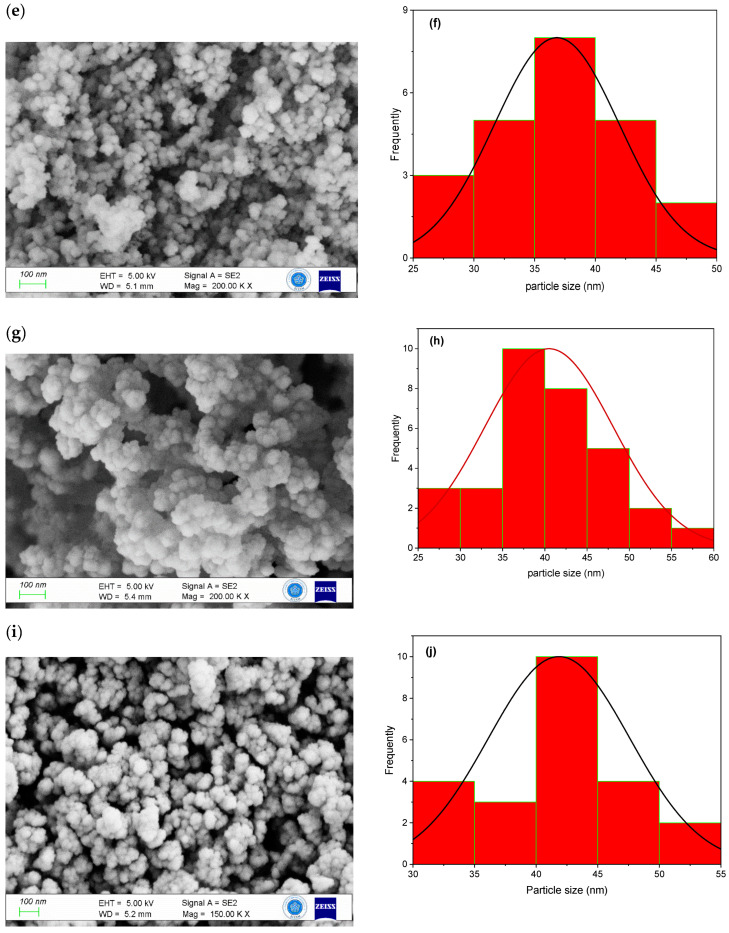
SEM micrographs and size distributions of MSNs prepared from sodium silicate obtained with different PEG concentration (**a**,**b**) 0.55, (**c**,**d**) 1.1 (**e**,**f**) 1.38 (**g**,**h**), 1.65 (**i**,**j**), and 2.2 mM.

SEM images reveal that the MSNs exhibit spherical to cotton ball-like particles with an average diameter ranging from 20 to 60 nm (Figure 6a–j). The addition of sodium silicate into a PEG/CTAC solution appears to promote silica agglomeration, facilitated by PEG. Silica wraps around the PEG/CTAC template, giving rise to spherical particles. In the presence of CTAC, variations in PEG concentration do not influence MSN shape or average particle size; nevertheless, at elevated PEG concentrations, particle size fluctuates from 35 nm to 43 nm, as illustrated in histograms (Figure 6b,d,f,h,j). Previous studies [57,58] have shown similar outcomes, indicating that increasing the PEG or NH_4_OH concentration decreases the particle size of silica nanoparticles. When utilizing ammonia as a catalyst, the average particle size of silica nanoparticles ranged from 140 nm to 29 nm as the pH shifted from 11 to 10.5. Primary mesoporous silica nanoparticles were acquired at pH 9.8, with sizes varying from 20 to 50 nm, in the presence of a non-ionic polyethylene oxide surfactant (Triton X100) acting as a structure-directing agent [30].

### 2.2. Adsorption Isotherm Study

**Figure 7:**
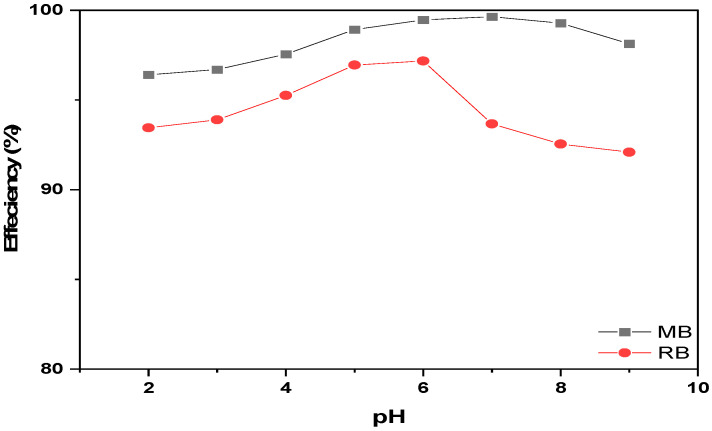
The effect of pH vale on the adsorption efficiency of dye on MSNs.

The adsorption capacity of the obtained MSNs at a 1.25 PEG/CTAC molar ratio which exhibited maximum surface area was evaluated with methylene blue and Rhodamine B dyes as probes. The ionization of silanol groups on the pore wall of the MSNs-OH adsorbent in aqueous solution (pH = 7) generates negatively charged species. Wang et al. proposed that the electrostatic attraction between MSNs and cationic MB or RB enhances extraction efficiency, alongside van der Waals forces. Consequently, MSNs exhibit a strong affinity for cationic dyes and demonstrate remarkable adsorption efficiency [59]. To assess the potential use of the synthesized materials as solid-phase sorbents for extracting and purifying dyes from wastewater, the maximum adsorption capacity and sorption rate were determined. The adsorption isotherms and kinetics of MB and RB on MSNs were extensively examined, along with analyzing the impact of pH on dye adsorption. Figure 7 illustrates the variation in adsorption capacity (*q_e_*) with increasing pH from 2 to 9. Initially, the adsorption capacity rises, peaking at pH 5 for RB and pH 7 for MB. Beyond these optimal pH values, the adsorption capacity declines due to the competition from hydroxyl ions in the solution.

**Figure 8:**
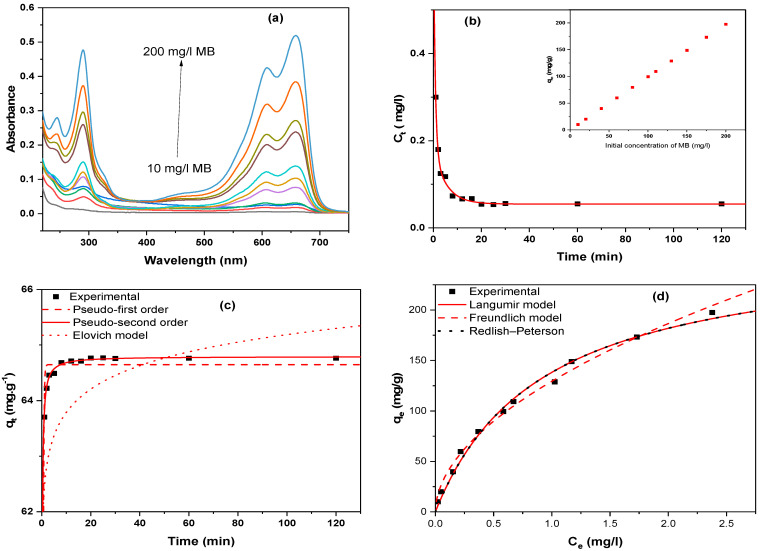
Experimental data and fitting models for MB: (**a**) UV-Vis spectra for initial concentration-dependent MB adsorption in the presence of mesoporous silica. (**b**) The residual concentration of MB in the solution at various times. (**c**) Experimental dynamic data for MB adsorption on MSNs, as well as typical nonlinear fitting data for the Elovich, pseudo-first order, and pseudo-second order models. (**d**) Experimental data for MSNs isothermal adsorption at various MB starting concentrations, as well as a nonlinear fitted curve based on Langmiur, Freundilich, and Redlich-Peterson isotherms (m = 100 mg, T = 25 °C, V_0_ = 100 mL, t = 60 min).

**Figure 9:**
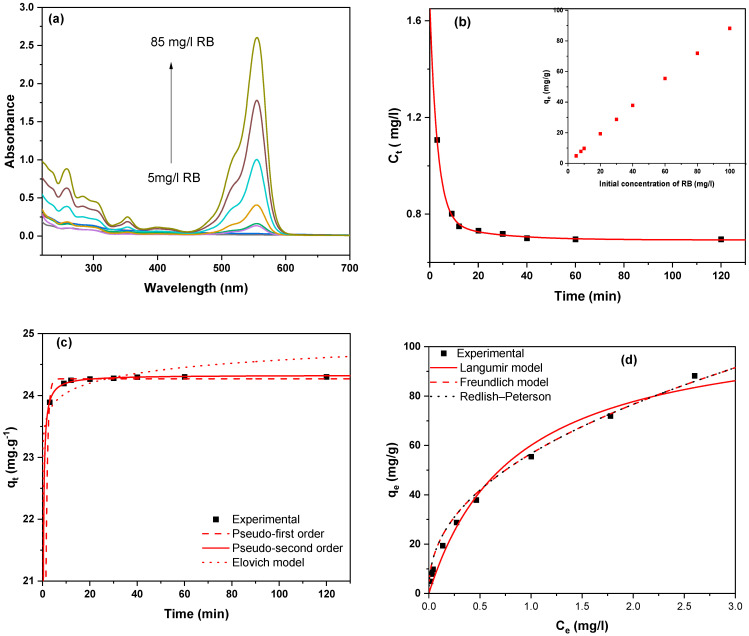
Experimental data and fitting models for RB: (**a**) Depicts UV-Vis spectra for initial concentration-dependent RB adsorption in the presence of mesoporous silica. (**b**) The residual concentration of MB in the solution at various times. (**c**) Experimental dynamic data for RB adsorption on MSNs, as well as typical nonlinear fitting data for the Elovich, pseudo-first order, and pseudo-second order models. (**d**) Experimental data for MSNs isothermal adsorption at various RB starting concentrations, as well as a nonlinear fitted curve based on Langmiur, Freundilich, and Redlich-Peterson isotherms (m = 100 mg, T = 25 °C, V_0_ = 100 mL, t = 60 min).

Figure 8a and Figure 9a show the changes in sorption spectra that correspond to MB and RB equilibrium concentrations in the presence of 100 mg of MSNs at different initial concentrations after 60 min. The typical adsorption intensity of MB (661 nm) and RB (545 nm) decreased dramatically after 10 min, showing that the residual concentration of dye in the solution decreases rapidly and the sorption effectiveness increases considerably over the short adsorption time (Figure 8b and Figure 9b). MB and RB clearance rates are approximately 90% during the first five minutes, showing a strong interaction between cationic dyes and MSN samples. Evidently, the adsorption reaches equilibrium after 60 min. The insets in Figure 8b and Figure 9b demonstrate that when the initial concentration increases, the equilibrium adsorptions (*q_e_*) continuously grow. Figure 8c and Figure 9c display experimental dynamic and typical fitting data (Elovich, pseudo-first sequential order, and pseudo-second order models) [60,61,62,63]. Table 2 lists the relevant parameters. The removal rate is clearly high, especially in the early stages, which is consistent with the removal efficiency results. This adsorption process is also compatible with the chemisorption pseudo second order model, as evidenced by the large coefficient of determination (*r*^2^ = 0.99). These could be attributable to the newly formed mesoporous structure, which enhances mass diffusion in addition to the electrostatic interaction between negatively charged silicate surfaces and positively charged dyes. Therefore, MSNs show potential applications as solid phase sorbent for the extraction of dyes either in analytical process or for large-scale treatment of dye-containing wastewater.

Adsorption capacity, which is defined as the mass of absorbent necessary to concentrate an adsorbate from a specific solution, is an important factor in processes requiring sorption. Figure 8d and Figure 9d illustrate that the equilibrium adsorption capacity increases with the initial concentration of cation dyes, resulting in an enhanced mass transfer driving force and increased diffusion at higher initial concentrations. To investigate MB and RB uptake on MSNs, three models (Langmiur, Freundilich, and Redlich-Peterson) [64,65,66,67] are employed. Table 3 outlines the parameters of these models. This procedure appears to be consistent with the Langmiur model, then the Redlich-Peterson isotherm. MB has a higher maximum adsorption capacity (*q_max_* = 256 mg g^−1^) than RB (*q_max_* = 110.3 mg g^−1^). The Redlich-Peterson isotherm equation for MB uses a constant value β of 1.01, which is about equivalent to 1. This indicated that the monolayer reaction of MB on MSNs was dominant. These findings suggest that MSNs with a wide surface area and constant pore width have cationic dye adsorption characteristics.

Table 4 compares the maximum adsorption capacities of the prepared MSNs with other adsorbents documented in the literature [60,68,69,70,71] for removal of RB and MB. In contrast to the other evaluated adsorbents, RB and MB exhibited a moderate maximum adsorption capacity (*q_max_*) on the prepared MSNs materials. Previous study [59] indicated that calcined SMS materials exhibited higher adsorption capacities for RB compared to the MSNs synthesized in in this work. The application of tetraethyl orthosilicate (TEOS) throughout the synthesis process may have modified the synthesis technique. Interestingly, the MSNs in this investigation exhibited superior MB adsorption capabilities compared to those documented in previous research.

## 3. Conclusions

The hydrothermally assisted sol-gel method developed in this study has enabled the synthesis of monodispersed MSNs with a narrow particle size distribution and adjustable pore dimensions. The utilization of PEG and CTAC as SDAs played a vital role in generating mesoporous particles with diverse morphologies ranging from cotton-like to spherical. These MSNs displayed a substantial surface area, rendering them suitable for use as solid-phase nano adsorbents in extraction and purification processes. The MSNs demonstrated remarkable adsorption efficiency towards cationic dyes, achieving extraction efficiencies exceeding 90% for the dyes tested in aqueous solutions. Adsorption experiments validated their effectiveness as adsorbents for MB and RB, demonstrating maximum adsorption capacities of 256 mg/g and 110.3 mg/g at pH 7 and 5, respectively. The adsorption isotherms were best described by the Langmuir model, followed by the Freundlich and Redlich–Peterson models, with adsorption kinetics following the pseudo-second-order model. These results highlight the potential of the synthesized MSNs as efficient nanosorbents for the solid-phase extraction and enrichment of dyes from complex matrices, presenting promising applications in sample preparation processes and in the remediation of dye-contaminated wastewater.

## 4. Materials and Methods

### 4.1. Synthesis of MSNs

MSNs particles with different sizes and morphologies were prepared from sodium silicate solution at various concentrations of PEG (0.55, 1.1, 1.38, 1.65, and 2.2 mM) using NH_4_OH as a catalyst (pH 10.5). In 100 mL flask, an aliquot of the commercial sodium silicate solution (SSS) was diluted with water to the concentration of 0.2 M and then pH was adjusted to 9 by drops of HNO_3_ (2 M). A 50 mL of diluted SSS was passed through cation exchange resin column to remove sodium and other cations. In another 100 mL flask, PEG and 0.053 g of CTAC were dissolved with 50 mL of water mixture containing NH_4_OH (3.0 mL) solution at 40–50 °C. The pH value should be adjusted to 10.5 with ammonia solution, where the total volume of reaction solution should be 150 mL. Subsequently, 50 mL of the SSS solution was added in a dropwise manner while solution stirred at 300 rpm for an hour. The components were subsequently transferred into an autoclave which was then sealed and placed into the oven for 12 h at 120 °C. The white precipitate was separated by centrifugation (500 rpm, 10 min) and washed by re-dispersion in deionized water and ethanol several times. Calcination at a temperature of 550 °C was carried out to remove the organic templates.

### 4.2. Characterization

Field emission scanning electron microscopy (FESEM) pictures were acquired utilizing a Toscana Zeiss-EM10C-100 KV (Oberkochen, Germany), and the average particle size was determined utilizing ImageJ 1.54g software. Nitrogen adsorption experiments were conducted at 77 K with a Micrometrics Model ASAP-2020 (Micromeritics, Norcross, GA, USA). The linear segment of the BET plot provided the specific surface areas (SBET) of the samples, while the Barrett-Joyner-Halenda method was employed to determine pore size distributions. The Fourier Transform Infrared Spectrometer (Thermo Fisher Scientific Inc., Waltham, MA, USA) and thermogravimetric analyser (NEXTA STA200, Hitachi, Japan) were utilized to obtain infrared spectra and evaluate weight loss as a function of temperature, separately. The dye concentrations in the experiments were quantified using a UV/visible spectrophotometer (Shimadzu UV-1800, Kyoto, Japan). This study use conventional wavelengths of 554 nm for Rhodamine B and 661 nm for Methylene Blue, due to their optimal molar absorptivity and consistent linear correlation between intensity and dye concentration.

### 4.3. Adsorption Study

The capability of MSNs to absorb (extract) the examined organic dyes were evaluated making use of batch mode experiments. The adsorption equilibrium capacity, *q_e_* (mg g^−1^) determined using solution containing 100 mg of the dispersed nano adsorbent, and 100 mL of the required adsorbate concentration was shaken for 60 min. The values of *q_e_* and efficiency (*η*) were computed from Equations (1) and (2).

(1) $$q_{e}=\frac{\left(C_{i}-C_{e}\right)\;V}{m}$$

where *V* is the volume of solution and *m* is the mass of adsorbent (g). The initial and equilibrium concentrations express by *C_i_* and *C_e_*, respectively.

(2) $$\%\;\eta =\left[\frac{C_{i}-C_{e}}{C_{i}}\right]\times 100$$

The impact of pH on the adsorption of methylene blue (MB) and rhodamine B (RB) onto MSNs was investigated to establish the optimal pH value for better extraction efficiency. The pH was examined over the range of 2 to 10 in aqueous solution, with initial acidity adjusted using sodium hydroxide and hydrochloric acid. The sorption rate of the adsorption process was determined by fitting the contact time data to the pseudo-first-order (Lagergren), pseudo-second-order, and Elovich models, as established in the Equations (3)–(5).

(3) $$q_{t}=q_{e}\;\left(1-e^{k_{1}t}\right)$$

(4) $$q_{t}=\frac{q_{e}^{2}k_{2}\;t}{{1+q_{t}\;k}_{2}t\;}$$

(5) qt=1b ln⁡(α b t+ 1)

where *q_t_* and *q_e_* (mg g^−1^) represent the adsorption capacity at different intervals (*t*) and equilibrium, respectively. *K*_1_ (min^−1^), *K*_2_ (g mg^−1^ min^−1^), *b*, and *α* are the rate constant for pseudo-first order, pseudo-second order, initial adsorption rate, and desorption constant, respectively.

Adsorption models (Langmuir, Freundlich, and Redlich-Peterson) employ isotherm data to determine the appropriate adsorption strategy. According to the Langmuir model (Equation (6)), molecules adsorb uniformly on the adsorbent’s surface without making contact with one another (monolayer sorption). According to the Freundlich isotherm (Equation (7)), sorption takes place on the adsorbent’s surface with varying energy. The adsorbed molecules interact, and the amount of solute adsorbed rises exponentially with concentration. The Redlich-Peterson model (Equation (8)) employs Langmuir’s equation as a limit for high concentrations and Freundlich’s for low concentrations.

(6) $$q_{e}=\frac{q_{max}{\;\;K}_{L}C_{e}}{1+\;K_{L}\;C_{e}}$$

(7) $$q_{e=K_{F\;}\;C_{e}^{1/n}\;}$$

(8) $$q_{e}=\frac{{\;\;K}_{r}C_{e}}{1+\;\alpha_{r}\;\;C_{e}^{\beta }\;\;}$$

where *K_L_* (L mg^−1^), *q_max_* (mg g^−1^), *K_F_* (mg g^−1^(L mg)^1/n^, and *K_r_* are the constant of the model about adsorption energy, maximum adsorption capacity of adsorbent, Freundlich adsorption, and Redlich-Peterson constants, respectively. *n*, *α_r_*, and *β* are is the adsorption intensity, Redlich-Peterson parameter, exponential constant, respectively.

## Figures and Tables

**Table 1:** BET specific surface area, BJH adsorption cumulative volume of pores and most probable pore diameter of samples synthesized at different weight ratios of PEG/CTAC ratio and constant sodium silicate, NH_4_OH, and water concentrations.

Sample	PEG (mM)	PEG/ CTAC Molar Ratio	Surface Area (m^2^/g)	Volume of Pores (cm^3^/g)	Most Probable Pore Diameter (nm)	Particle Size (nm)
MSNs-1	0.55	0.5	633.30	2.1	2.4	31
MSNs-2	1.10	1	759.98	1.7	2.8	37
MSNs-3	1.38	1.25	1011.42	2.4	3.1	41
MSNs-4	1.65	1.5	779.31	2.3	2.8	43
MSNs-5	2.2	2	711.22	1.9	2.8	35

Note: The pH value of reaction system is 10.5, and the hydrothermal condition is 9 h at 120 °C.

**Table 2:** Kinetic parameters for three kinetic models in the MB and RB adsorption on the MSNs.

Kinetic Models	Parameters	MB	RB
Pseudo-first order model	*q_e_* (mg g^−1^)	64.65 ± 5	24.27 ± 3
	*K*_1_ (min^−1^)	4.2 ± 0.5	1.36 ± 0.4
	*r* ^2^	0.73	9.34
Pseudo-second order model	*q_e_* (mg g^−1^)	64.79 ± 4	24.33 ± 2
	*K*_2_ (g mg^−1^ min^−1^)	0.89 ± 0.03	0.76 ±0.03
	*r* ^2^	0.99	0.99
Elovich model	*α* (mg g ^−1^ min^−1^)	2.14 × 10^4^ ± 600	5.8 × 10^3^ ± 400
	*b* (g mg^−1^)	1.64 ± 0.4	4.34 ± 0.2
	*r* ^2^	0.93	0.51

**Table 3:** Isotherm parameters for three models in the MB and RB adsorption on the MSNs.

Adsorption Isotherms	Parameters	MB	RB
Langmuir	*q_max_* (mg g^−1^)	256.27 ± 10	110.37 ± 4
	*K_L_* (L mg^−1^)	1.09 ± 0.1	1.19 ± 0.2
	*r* ^2^	0.99	0.983
Freundlich	*K_f_* (L mg^−1^)^1/*n*^ (mg g^−1^)	129.43 ± 5	56.68 ± 3
	*n*	1.89 ± 0.2	2.29 ± 0.25
	*r* ^2^	0.991	0.973
Redlish–Peterson	*K_R_* (L/g)	288.95 ± 15	1.7 × 10^5^± 500
	*α_r_* (L/mg)	1.09 ± 0.1	3002 ± 25
	*β*	1 ± 0.1	0.56 ± 0.05
	*r* ^2^	0.97	0.999

**Table 4:** Comparison of the q_max_ of MB and RB on different silica nanoparticle absorbents.

Adsorbent	MB	RB	Reference
	*q_max_* (mg/g)	*q_max_* (mg/g) or %	
MCM-41		1.35	[68]
MCM-41 Calcined		1.52	[68]
MCM-41	54		[69]
SMS (reversed MCM-41)		287.4	[70]
SBA-15	45.08		[71]
HMS-OH	165.1	90%	[59]
MS-C		131	[60]
C-MS-C		72	[60]
MSNs-3	256	110.3	This study

Mobile composition of matter (MCM); Santa Barbara amorphous (SBA-15); Spherical mesoporous silica (SMS) reversed MCM-41; Mesoporous silica with wormhole framework structure (HMS-OH); Ordered mesoporous silica cubic particles (MS-C); C-MS-C calcined ordered mesoporous silica cubic particles.

## Data Availability

The data are contained within the article.
